# Parental anxiety related to referral of childhood heart murmur; an observational/interventional study

**DOI:** 10.1186/s12887-015-0507-4

**Published:** 2015-11-21

**Authors:** Tonje Bårdsen, Mari Hoven Sørbye, Håvard Trønnes, Gottfried Greve, Ansgar Berg

**Affiliations:** Department of Clinical Science, University of Bergen, Bergen, Norway; Department of Global Public Health and Primary Care, University of Bergen, Bergen, Norway; Department of Paediatrics, Haukeland University Hospital, Bergen, Norway; Department of Heart Disease, Haukeland University Hospital, Bergen, Norway

**Keywords:** Cardiology, Childhood, Heart murmur, Parental anxiety, Fact sheet

## Abstract

**Background:**

Detection of a heart murmur in healthy children is common, but may generate anxiety among parents. Many parents believe a heart murmur is a sign of heart disease, although the majority of heart murmurs are innocent. The purpose of this study was to assess anxiety and concerns in parents of children referred for evaluation of a heart murmur and to evaluate the effect of receiving a fact sheet about heart murmurs before the cardiologic consultation.

**Methods:**

Parents of children referred for evaluation of a heart murmur responded to questionnaires assessing family and patient characteristics, parental concerns and anxiety. Anxiety was measured using the State Trait Anxiety Index (STAI) before and two weeks after the consultation. One third of the parents received a fact sheet before the consultation.

**Results:**

Two hundred fifty-eight parents of 178 children participated. About 60 % of the parents had an increased level of anxiety before the consultation. The majority of the parents (71 %) had at least one major concern about heart murmurs in children, and having a concern was related to higher anxiety levels (*p* = 0.02). Anxious personality and lower education predicted an increased anxiety level. Before the consultation, parents who received a fact sheet presented a lower mean STAI state anxiety level (33.2) than those who did not (35.3), but the difference was not significant (*p* = 0.09). Fewer parents in the intervention group believed their child would have increased risk of heart disease later in life (*p* = 0.04) or that heart murmurs in children represents valvular-or congenital heart disease (*p* = 0.02). After the consultation, parental anxiety decreased from a mean STAI state of 34.9 to 30.6 (*p* < 0.01), and the mean STAI state scores were similar for the control and intervention group.

**Conclusion:**

Parents with a child referred for a heart murmur presented a higher mean anxiety level than pre-school parents, and having an anxious personality, a major concern or low education predicted an increased anxiety level. After the consultation, parental anxiety decreased. Receiving a fact sheet about heart murmurs did not significantly reduce parental anxiety levels, but had a modest effect on concerns for the consequences of a heart murmur.

**Electronic supplementary material:**

The online version of this article (doi:10.1186/s12887-015-0507-4) contains supplementary material, which is available to authorized users.

## Background

Detection of a heart murmur in otherwise healthy children is common, but may generate parental anxiety. Many parents deem a heart murmur as a sign of structural heart abnormality despite the fact that most childhood heart murmurs are innocent and do not cause morbidity [1–7]. Previous studies have shown that parental concern is common and that parents may interpret a heart murmur as a condition that most likely implicates medication, surgery or restriction of physical activity [1, 2, 6]. We have in a previous study shown that only 10 % of Norwegian children referred to a paediatric cardiologist for evaluation of a heart murmur needed follow-up, medication or surgery [8]. Still, unwarranted parental anxiety may have consequences for the child. Bergman and Stamm [6] found that 40 % of healthy children whose parents at one point had perceived that their child had “something wrong with their heart” were restricted in some way.

Several studies have identified predictors for parental anxiety in general and parental anxiety related to referral for a heart murmur, including low education [3, 6, 9, 10], an anxious personality [1], being a mother [2, 9–11], not knowing what the term “heart murmur” means [7] and previous family history of heart disease [6], but few have assessed if parental concerns for the implications of a heart murmur (hereafter called “major concerns”) predict anxiety [7].

It has been shown that examination and information by a paediatric cardiologist is an effective measure to ease anxiety and clarify misconceptions [1–4, 7], although some parents have persisting anxiety [1–5, 7]. A fact sheet with information about heart murmurs may contribute to ease parental anxiety as well as relieve major concerns, but to our knowledge, this has not previously been properly evaluated.

Our primary aims were to assess the level of anxiety among parents with a child referred for cardiologic examination of a heart murmur and to evaluate the effect of a fact sheet on anxiety levels. The secondary aims were to assess major concerns among the parents and to explore possible predictors for having an increased level of anxiety. We hypothesised that major concerns about heart murmurs increase anxiety and that parental concerns and anxiety levels would be reduced after receiving a written fact sheet about childhood heart murmurs compared to the control group.

## Methods

### Study design

The design of this study was part observational and part interventional. In the observational part, we explored anxiety levels using the State-Trait Anxiety Inventory (STAI), version X1, before and two weeks after the consultation. Parental concerns were assessed by different questions about the cause and consequences of heart murmurs in children. We also investigated possible predictors for increased anxiety levels and concerns. The intervention was a fact sheet on heart murmurs in children (Additional file 1), which was sent to the last one third of the parents (*n* = 70). Since all children underwent a cardiologic evaluation including an echocardiogram, this was not considered as an intervention.

### Study population

**Fig. 1:**
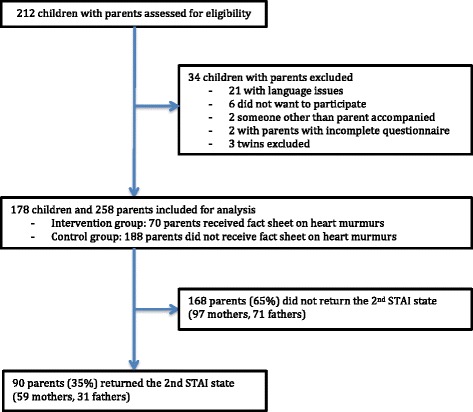
Flow chart of inclusion of study group, intervention and response rate

All participants were recruited from the Outpatient Clinic at Haukeland University Hospital in Bergen, Norway in the period February 21^st^ 2012 to October 29^th^ 2013. The study subjects consisted of parents of children referred to a paediatric cardiologist for a first time evaluation of a heart murmur without other signs of cardiologic disease. Parents were invited to participate in the study if they were able to communicate and read Norwegian. If both parents were present at the consultation, both were included. We excluded parents who returned an incomplete questionnaire. Parents with more than one child referred for evaluation of a heart murmur were included only once. Inclusion of parents is shown in Fig. 1. All children were referred from a general practitioner or a paediatrician and were examined clinically and with echocardiography by an experienced paediatric cardiologist, to rule out the presence of a structural heart defect and to reassure the parents. All parents gave informed, written consent. The study was approved by the “Western Regional Ethics Committee, Norway”, the regional committee for medical and health research ethics.

### Data collection

Parental anxiety was assessed with the STAI in two questionnaires given to the parents before the consultation: one to complete prior to the consultation and the other 1–2 weeks later. In the first questionnaire we also assessed parental concern with different questions, and parents reported socio-demographic data and if they had a family history of heart murmurs or heart disease (Additional file 2).

Information on the children’s demographic data and previous disease that caused hospitalisation was obtained from their hospital records. From this source, we also acquired the waiting time from referral to consultation, if the referring doctor was a general practitioner or paediatrician and the final diagnosis after the consultation.

The fact sheet was mailed to the last third of the recruited parents two weeks prior to the consultation. The fact sheet presented the most common causes of heart murmurs in children and emphasised the low risk of having a serious heart condition with an isolated heart murmur (Additional file 1).

### Outcomes

The primary outcomes of this study were anxiety levels measured by the STAI state [12], including anxiety levels before and after the consultation as well as with or without intervention. The STAI is a self-reporting questionnaire consisting of two parts: the STAI state and the STAI trait. The STAI state assesses anxiety at a particular moment in time and therefore it will vary depending on the situation. The STAI trait assesses general anxiety in a person and therefore it will be relatively stable over time, and independent of the situation. It is a validated and acknowledged tool for assessing anxiety in different populations and languages [12] and has been used in several studies on parental anxiety related to childhood heart murmurs and other health conditions in children [1, 7, 9–11, 13]. Each questionnaire consists of 20 statements with a scale ranging from 1 to 4. The level of anxiety is measured with scores ranging from 20 to 80 with higher scores correlating with higher levels of anxiety. We used a Norwegian version of the STAI, translated and validated by K. Håseth [14]. We assessed the STAI state level before and two weeks after the consultation. We also compared the STAI state levels between the control group who did not receive a fact sheet and the intervention group who did.

The secondary outcomes of this study were parent’s major concerns on heart murmurs and what parents believed to be the most common cause of heart murmurs in children. We assessed parental concerns about heart murmurs with different questions regarding the relation between childhood heart murmurs and heart disease (Additional file 2). The questions were similar to statements used in previous studies [1–3], but they have not been validated. The questions were the following:

1. *What impact do you believe the heart murmur will have on your child’s activity?*
2. *How serious is it to have a physiologic heart murmur?*
3. *To what degree do you believe your child has increased risk of heart disease later in life?*

The parents were asked to grade the different questions from 1 to 5 on a Visual Analogue Scale (VAS) with anchors (not at all-very much). The cut-off point for having a major concern was set to a score higher than 1.5 on the VAS, this point being measured with a ruler. The parents were also asked what they thought was the most common cause of heart murmurs in children, with the options being “natural phenomenon”, “valvular heart disease”, “heart attack”, or “congenital heart disease”. If the parent responded other than “natural phenomenon”, it was also considered a major concern.

We considered socio-demographic factors, low education (12 years of education or less), previous hospitalisation of the child, family history of heart murmur or heart disease, major concerns about heart murmurs, long wait time and being referred by a general practitioner as opposed to a specialist as potential predictors for having an increased level of anxiety. We wanted to investigate the effect of seeking information on heart murmurs beforehand and how that would influence anxiety levels, and if there was a difference on the effect depending on the source (internet, friends/family, encyclopaedias or newspapers).

### Data analysis

When comparing STAI state scores of different groups we used the variable as continuous. Comparisons of STAI state scores between mothers and fathers, responders and non-responders and control and intervention group, were carried out with Mann–Whitney *U* test. We used Wilcoxon signed-rank test to evaluate the parents’ STAI scores before and after the cardiologic consultation. Comparisons of concerns in the control group and in the intervention group were performed with Chi-square tests.

We used a binary logistic regression model to identify potential predictors for increased anxiety levels. The STAI state scores were not normally distributed (Shapiro-Wilk statistic =0.952, *P* value <0.01), and were therefore used as a binary variable for this analysis. To our knowledge, there is no STAI state norm for parents of young children. However, a previous study reported a mean STAI state score of 31 for Norwegian parents of preschool children [11]. Since this study group was relatively comparable to ours, we defined a STAI state score of 31 as a cut-off point. Parents with a STAI state score less than or equal to 31 were defined as having a decreased level of anxiety and those with a score over 31 were defined as having an increased level.

Since more than one parent per child were included in the study group, some observations were not entirely independent. Therefore, we repeated all analyses with inclusion of only one parent per child. All statistical tests were two-sided with a significance level of 5 % and were performed with the use of SPSS statistics, version 20.

## Results

### Study population

Of 212 children who met with their parents for evaluation of an isolated heart murmur, a total of 258 parents of 178 children were included (Fig. 1). Of these, 90 parents (35 %) returned the second STAI questionnaire. Among the 70 parents who received a fact sheet about heart murmurs in children before the appointment, 27 parents (39 %) returned the second questionnaire. Characteristics of the study population are outlined in Table 1. The children were almost equally divided between boys and girls. The mean age was 3.5 years with an age range from 3 months to 15 years. There were no large differences in parent and child characteristics, STAI scores between those who returned the second STAI questionnaire and those who did not (Additional file 3) or between the control and the intervention group (Additional file 4).

**Table 1:** Characteristics of parents and children participating in the study

Characteristics	Numbers (percent)
Parents *n* = 258	
Female gender	156 (61 %)
Both parents live with child	227 (88 %)
More than 12 years of education	148 (59 %)
Parent with more than one child	192 (74 %)
Family history of HD	64 (25 %)
Family history of HM	64 (25 %)
Looked up information on HM	113 (44 %)
Children *n* = 178	
Age (years, mean ± SD)	3.50 ± 3.03
Previous hospitalisation	55 (31 %)
General info	
Waiting time for consultation (months, mean ± SD)	2.50 ± 0.98
Referred by a specialist	40 (16 %)
Returned the second STAI questionnaire	90 (35 %)
Received informational sheet	70 (27 %)

*Abbreviations: HD* Heart disease, *HM* Heart murmur, *SD* Standard deviation, *STAI* State trait anxiety inventory

### Anxiety level

The final STAI state and trait scores were obtained following the STAI manual [12]. Before the consultation, 42 % of the parents were in the decreased anxiety group (STAI state score ≤31) and 58 % in the increased anxiety group (STAI state score >31). The mean STAI trait and STAI state score of all parents before the appointment were 37.6 and 34.7, respectively. The mothers had a significantly higher mean STAI state score than the fathers (36.0 and 32.8, respectively, *p* = 0.03). The mean STAI trait score of the mothers (38.8) was also significantly higher than the fathers’ (35.8) (*p* = 0.01). After restricting the analysis to include only one parent per child, the gender difference in mean STAI state score diminished, while the difference in STAI trait score remained unchanged.

Parents who received a fact sheet before the consultation had a lower level of anxiety (mean 33.2) than the control group (mean 35.3), but the difference was not significant (*p* = 0.09). Overall parental anxiety was significantly lower after the cardiologic examination, with a decrease in mean STAI state score from 34.9 to 30.7 (*p* < 0.01, Table 2). The post-consultation STAI scores were similar for those who received or did not receive a fact sheet (Table 2). Restricting the analysis to only one parent per child did not substantially change the overall STAI state scores, and the comparison of the control and the intervention group was not meaningful due to small numbers.

**Table 2:** Parental STAI state scores of responders (*n* = 90) before and after the consultation, Wilcoxon signed rank test

STAI State (mean ± SD)	Before consultation	After consultation	*P*-value
All *n* = 90	34.93 ± 9.57	30.67 ± 9.03	<0.01
Control *n* = 63	34.76 ± 9.84	30.75 ± 9.28	<0.01
Intervention *n* = 27	35.33 ± 9.23	30.48 ± 8.58	0.01

*Abbreviations: SD* Standard deviation, *STAI* State trait anxiety inventory

### Variables associated with increased anxiety

Predictors for an increased level of anxiety are displayed in Table 3. Parents who had major concerns about the implications of a heart murmur, with increased STAI trait scores or low education were more likely to have an increased STAI state score. Seeking information on heart murmurs beforehand, regardless the source, presented neither a lower nor a higher anxiety level than the others’. Furthermore, a family history of heart murmurs or heart disease, or previous hospitalisation of the child did not affect the anxiety level. Parental anxiety was not influenced by the wait time or if the referring doctor was a specialist. The estimates were practically similar after restricting the analyses to only one parent per child.

**Table 3:** Risk factors for an increased level of anxiety in parents (STAI score >31)

Characteristics	Odds ratio	95 % CI for OR	*P*-value
Parent			
STAI trait	1.24^a^	1.18–1.31	<0.01
Female gender	1.25	0.75–2.06	0.39
12 years education or less	2.53	1.49–4.32	<0.01
Parents live separately	1.87	0.79–4.41	0.16
Parent with only one child	1.17	0.64–2.12	0.61
Family history of HD	1.03	0.58–1.83	0.93
Family history of HM	0.92	0.54–1.56	0.75
Sought information on HM	1.49	0.90–2.46	0.12
Child			
Age over 2 years	0.96	0.58–1.58	0.86
Previous hospitalisation	1.13	0.65–1.96	0.66
General info			
Waiting time for consultation >2 months	1.02	0.62–1.67	0.95
Referred by a specialist	0.75	0.38–1.49	0.42
Did not received a fact sheet	1.46	0.84–2.53	0.18
Returned STAI after appointment	1.13	0.67–1.89	0.66
Concerns			
It is serious to have a physiological HM	2.72	1.59–4.65	<0.01
HM will restrict the Child’s activity	3.13	1.65–5.91	<0.01
HM will increase risk of HD later in life	2.72	1.60–4.63	<0.01
Most likely cause of HM in children is CDH	1.75	1.01–3.03	0.05
Has a major concern	2.54	1.47–4.38	<0.01

*Abbreviations: CHD* Congenital Heart disease, *HD* Heart disease, *HM* Heart murmur, *SD* Standard deviation, *STAI* State trait anxiety inventory

^a^Odds ratio with increment of 1 STAI-trait score

### Major concerns

Most parents (71 %) in our study had at least one major concern about their child’s heart murmur. The most common concern was that this finding probably represented serious illness (57 %). Half of the parents (50 %) believed that their child would be at increased risk of heart disease later in life. Approximately one third (34 %) of the parents answered that a heart murmur is most likely caused by valvular-or congenital heart disease, and 26 % thought that the presence of a heart murmur would result in restriction of their child’s physical activity. Analysis with only one parent per child showed similar results.

A comparison of the level of major concerns between the control and intervention group is presented in Table 4. Generally, the total level of major concerns was slightly lower in the intervention group (64 %) than in the control group (73 %), but the difference was not significant (*p* = 0.18). However, fewer parents in the intervention group believed that their child would have increased risk of heart disease later in life (*p* = 0.04) or that the most probable cause of heart murmur in children was valvular-or congenital heart disease (*p* = 0.02). Again, analysis of subgroups with restrictions of one parent per child provided no meaningful results.

**Table 4:** Comparing concerns in control group vs. intervention group. Parents have a major concern if score > 1.5 on VAS scale

Major concerns	Control group	Intervention group	*P* value
	*n* = 188	*n* = 70	
*It is serious to have a physiological heart murmur*	111 (59 %)	36 (51 %)	0.12
*The heart murmur will restrict the child’s activity*	48 (26 %)	18 (26 %)	0.85
*The child has increased risk of heart disease later in life*	101 (54 %)	28 (40 %)	0.04
*Most likely cause of heart murmur in children is structural heart disease*	71 (38 %)	16 (23 %)	0.02
Total			
Has a major concern	137 (73 %)	45 (64 %)	0.18

A total of 18 (10 %) children were in need of a follow-up appointment and 14 children (8 %) were diagnosed with a congenital heart disease.

## Discussion

The present study is the largest study that has assessed anxiety and concerns in parents of children referred for heart murmurs and the first to evaluate if a fact sheet about heart murmurs reduces anxiety and concerns in parents of children with a heart murmur.

We found that parents with a child referred for evaluation of a heart murmur presented an overall higher mean level of anxiety than previously reported for Norwegian parents of preschool children [11]. The parents who received an informational fact sheet on childhood heart murmurs had slightly, but not significantly, less concerns compared to the control group. There was no difference in anxiety levels. After the cardiologic consultation, anxiety levels were similar in both groups and significantly lower than before the consultation. This suggests that thorough examination of the child and oral information from a cardiologist is reassuring for parents of a child with a heart murmur, as previous studies have showed [1–4, 7], whereas a fact sheet does not provide an additional benefit.

Predictors of increased anxiety levels were having major concerns about paediatric heart murmurs, an anxious personality, and low education. The association between low education and increased STAI state score has also been found in other studies [3, 9, 10]. In line with the STAI manual, an increased STAI trait score predicted an increased STAI state score [12]. A possible explanation is that people with anxious personalities are prone to respond to a stressful situation with anxiety. Unexpectedly, having a family history of heart disease or heart murmur did not influence the level of anxiety. We expected to find higher anxiety levels among parents with a family history of heart disease as shown by Bergman and Stamm [6], and lower anxiety levels among parents with family history of heart murmurs. Previous studies have reported that mothers have a significantly higher level of anxiety than fathers [2, 9–11], which is in accordance with our findings.

In our study, only 56 % of the parents could identify that the most common cause of heart murmur in children is a natural phenomenon. In accordance with our hypothesis major concerns and misconceptions among parents were predictors for an increased anxiety level. Parents who sought information about heart murmurs before the consultation had the same anxiety levels and rate of major concerns as other parents, which may indicate that self-attained information may be difficult to interpret and does not offer reassurance. It is thus possible that providing reliable information about heart murmurs to parents would reduce misconceptions and thereby decrease parental anxiety. Scanlon [15] tested if a fact sheet about innocent heart murmurs could be educative towards a group of parents of healthy children. The intervention group showed significant decrease in the number of parents with “harmful understanding” and “anxious” responses concerning innocent heart murmurs compared to the control group [15]. In the present study, however, a fact sheet did not significantly reduce anxiety and only had a modest effect on the parents’ concerns.

Strengths of this study are a large sample size and an unselected study group. Our findings mostly correspond with previous studies from different countries [1–5, 15] and this suggests that the results may be generalised to different cultures and social groups. Our study group was quite evenly distributed regarding parental sex and education, whereas previous studies tend to have an overrepresentation of mothers [1, 2, 5, 7, 13]. Anxiety was assessed using the STAI-X1, which is a well-validated psychometric instrument that has been used in different populations, cultures and languages as well as in other studies measuring anxiety parental anxiety [1, 7, 9–14]. Our study was, to our knowledge, the first to evaluate the effect a fact sheet has on anxiety and concerns of parents with a child with a heart murmur.

An important weakness is the overall low response rate on the second STAI questionnaire (35 %). This has most likely hampered the power of the study, which may have led to a type II error. It is also possible that parents who were reassured were more likely to respond, resulting in a selection bias. If so, the observed reduction in STAI state score after the consultation may have been exaggerated. However, there were no substantial differences in characteristics or pre-consultation STAI-scores between responders and non-responders, suggesting that selection bias was less likely to be present. A weakness is also that the distribution of the fact sheets was not randomised, which theoretically could cause additional selection bias. Still, there was no reason to believe that parents of children referred in a specific period of time would differ from the control group. The selection of the intervention group may therefore be regarded as a proxy to randomisation. This notion is supported by the findings of similar characteristics in the two groups.

Another limitation is that more than one parent per child participated in almost half of the cases. This could have biased the results, since parents of the same child may present the same view. We pursued this possibility by restricting the analysis to one parent per child and found no large impact on the results. The statements regarding heart murmurs have not been validated, although variants of these have been used in previous studies [1–3]. Because of the lack of validation, the results concerning these statements must be interpreted with caution.

Setting a cut-off point of the STAI score also poses a challenge. The STAI score is a continuous variable, and it is therefore impossible to find an exact cut-off point that divides the population in two homogenous groups, as anxiety levels will vary in both groups. It is important to keep in mind that the STAI state score ranges from 20–80, and that a score >31 represents a higher score than the mean of parents of preschool children [11], and not necessarily a high level of anxiety. A certain level of anxiety may also be reasonable, since 8 % of the children in our study and 10 % in our previous study [8] were diagnosed with congenital heart disease.

## Conclusion

Parents with a child referred for a heart murmur presented a higher mean anxiety level than previously reported for pre-school parents, and having a major concern, an anxious personality and a low educational level predicted an increased anxiety level. Receiving a fact sheet on heart murmurs had a modest impact on parental concerns, but the overall anxiety level was not significantly reduced. In contrast, parental anxiety was significantly reduced after the cardiologic consultation. This confirms that cardiologic evaluation and information is an important measure for reassuring these parents and suggests that receiving a fact sheet provides no substantial benefit.

## Additional files

Additional file 1:
**Translated fact sheet: A translated version of the fact sheet that was distributed to 1/3 of the parents before the consultation.** (PDF 37 kb) Additional file 2:
**Translated questionnaire: the distributed questionnaire, translated from Norwegian.** (PDF 132 kb) Additional file 3:
**Comparison of characteristics between responders (** ***n*** **= 90) and non-responders (** ***n*** **= 168) of the second STAI questionnaire, table.** (PDF 68 kb) Additional file 4:
**Comparison of characteristics between control group (did not receive a fact sheet) and intervention group (received a fact sheet), table.** (PDF 66 kb)

## Abbreviations

STAI: State trait anxiety index
VAS: Visual analogue scale
