# Supplementary material for: Parental anxiety related to referral of childhood heart murmur; an observational/interventional study
Source: BMC Pediatr. 2015 Nov 21;15:193. doi: 10.1186/s12887-015-0507-4 (PMC4654835; doi:10.1186/s12887-015-0507-4)
Supplement: Additional file 1: — Translated fact sheet: A translated version of the fact sheet that was distributed to 1/3 of the parents before the consultation. (PDF 37 kb) [file 12887_2015_507_MOESM1_ESM.pdf]

Dear parents
Your child has been referred to Section of Paediatric Cardiology (Haukeland University Hospital) because there has been heard a heart murmur, or questioned if there is heart disease. Since this often seems frightening we want to give you some information about heart murmurs before the consultation.

The doctor who referred your child has heard a heart murmur on your child. Heart murmurs are very common and are present in 70-80% of healthy children without being a sign of heart disease. Most children with a heart murmur are healthy with a normal heart, and the murmur is an innocent sound phenomenon. This phenomenon is called “stills murmur” or “a physiologic murmur”. However, in rare cases the murmur may be due to minor structural heart defects (<1% of all children). It is not always easy to tell the innocent murmurs from those due to structural heart defects. That is what will be examined.

At the consultation we will examine your child and listen to the heart. Thereafter we will look at the heart with ultrasound. This is done in the same way as in prenatal care, the child receives gel on the chest and a sound transducer is placed on the chest. You as parents are present during the whole examination, which lasts approximately 30 minutes, but can last longer. Most children are comfortable during the ultrasound examination.

When the examination is done, we will discuss the findings with you. In most cases everything is normal, but if necessary, we will agree upon a follow up or further examinations.

Best regards

The Child Cardiologists
