# Supplementary material for: Parental anxiety related to referral of childhood heart murmur; an observational/interventional study
Source: BMC Pediatr. 2015 Nov 21;15:193. doi: 10.1186/s12887-015-0507-4 (PMC4654835; doi:10.1186/s12887-015-0507-4)
Supplement: Additional file 2: — Translated questionnaire: the distributed questionnaire, translated from Norwegian. (PDF 132 kb) [file 12887_2015_507_MOESM2_ESM.pdf]

Translated Questionnaire

On parent:

1) Mother □ Father □ Other______________________

2) Both parents live with the child □
Sole parental care □
Shared parental care □

3) *Highest level of education:* Elementary school □
High school □
University/college 1-4 years □
University/college over 4 years □

4) *Heart disease present in close family* (Your self, parents, siblings, partner) Yes□ No□

5) *Heart murmur present in close family?* (Your self, parents, child's siblings, partner): Yes□ No□
If yes, who: ____

On child:

6) *Birth order:*
No____ of total number of siblings: ___

General questions:

7) *Have you sought information on heart murmurs?* Yes□ No□

8) *If yes, where did you seek information?* Internet □
Friends and acquaintances□
Your parents/grand parents□
Newspapers□
Encyclopaedias□

9) *To what degree were you relieved by the information?*

Relieved — Considerably more concerned

1 — 5

10) *What impact do you believe the heart murmur will have on your child's physical activity?*

No impact — Considerably reduced physical activity

1 — 5

11) *How serious is it to have a physiological heart murmur?*

Completely harmless — Very Serious

1 — 5

12) *What do you believe is the most common cause of heart murmur in children?*
Valvular problem□
Congenital heart disease□
Heart attack□
Natural phenomenon□

13) *To what degree do you believe your child has increased risk of heart disease later in life?*

No risk High risk

1 **5**
