# Supplementary material for: Parental anxiety related to referral of childhood heart murmur; an observational/interventional study
Source: BMC Pediatr. 2015 Nov 21;15:193. doi: 10.1186/s12887-015-0507-4 (PMC4654835; doi:10.1186/s12887-015-0507-4)
Supplement: Additional file 3: — Comparison of characteristics between responders ( n = 90) and non-responders ( n = 168) of the second STAI questionnaire, table. (PDF 68 kb) [file 12887_2015_507_MOESM3_ESM.pdf]

Additional file 3

Comparison of characteristics between responders (n=90) and non-responders (n=168) of the second STAI Sheet

	Responder<br>n=90	Non responder<br>n=168
Gender (female)	59 (66%)	97 (58%)
Both parents live with child	81 (90%)	146 (87%)
Education level over 12 years	55 (61%)	93 (55%)
Has more than one child	68 (76%)	124 (74%)
Family history HD	22 (24%)	42 (25%)
Family history HM	22 (24%)	42 (25%)
Looked up info on HM	45 (50%)	68 (41%)
**Children**	**n=62**	**n=116**
Gender (female)	36 (58%)	56 (48%)
Age (years, mean±SD)	3.73 ± 3.5	3.38 ± 2.7
Only child	13 (21%)	23 (20%)
First born	29 (47%)	40 (35%)
Both parents accompany the child	29 (47%)	62 (53%)
Previous hospitalisation	17 (27%)	38 (33%)
Diagnosed with CHD	4 (6.5%)	10 (8.6%)
**General info**	**n=90**	**n=168**
Wait time (months, mean±SD)	2.43 ± 1.1	2.54 ± 0.9
Referred by a specialist	12 (13%)	28 (17%)
Received informational sheet	27 (30%)	43 (26%)
**STAI**		
STAI state (mean±SD)	34.9±9.6	34.6±9.8
STAI trait (mean±SD)	37.3±8.5	37.8±9.6
**Concern**		
Has a major concern	65 (72%)	117 (70%)
Serious to have a physiologic HM	56 (62%)	91 (54%)
Childs activity will be restricted of HM	22 (24%)	44 (26%)
Child increased risk of HD later	44 (49%)	85 (51%)
Most likely cause to HM in children is CHD	34 (38%)	53 (32%)

Abbreviations HM: heart murmur, HD: heart disease, CHD: congenital heart disease
