# Supplementary material for: Parental anxiety related to referral of childhood heart murmur; an observational/interventional study
Source: BMC Pediatr. 2015 Nov 21;15:193. doi: 10.1186/s12887-015-0507-4 (PMC4654835; doi:10.1186/s12887-015-0507-4)
Supplement: Additional file 4: — Comparison of characteristics between control group (did not receive a fact sheet) and intervention group (received a fact sheet), table. (PDF 66 kb) [file 12887_2015_507_MOESM4_ESM.pdf]

Additional file 4

Comparison of characteristics between Control group (did not receive a fact sheet) and Intervention group (received a fact sheet)

	Control group n=188	Intervention group n=70
Gender (female)	109 (58%)	47 (67%)
Both parents live with child	164 (87%)	63 (90%)
Education level over 12 years	102 (54%)	46 (66%)
Has more than one child	144 (77%)	48 (69%)
Family history HD	52 (28%)	12 (17%)
Family history HM	46 (25%)	18 (26%)
Looked up info on HM	79 (42%)	34 (49%)
**Children**	**n=129**	**n=49**
Gender (female)	68 (53%)	24 (49%)
Age (years, mean±SD)	3.82 ± 3.3	2.66 ±1.9
Only child	24 (19%)	12 (25%)
First born	47 (36%)	22 (45%)
Both parents accompany the child	64 (50%)	27 (55%)
Previous hospitalisation	38 (30%)	17 (35%)
Diagnosed with CHD	12 (9%)	2 (4%)
**General info**	**n=188**	**n=70**
Wait time (months, mean±SD)	2.57 ± 1.0	2.33 ± 0.9
Referred by a specialist	31 (17%)	9 (13%)
Received informational sheet	63 (34%)	27 (39%)
**STAI**		
STAI state (mean±SD)	35.3±9.8	33.2±9.2
STAI trait (mean±SD)	38.2±9.4	36.0±8.6
**Concern**		
Has a major concern	137 (73%)	45 (64%)
Serious to have a physiologic HM	111 (59%)	36 (51%)
Childs activity will be restricted of HM	48 (26%)	18 (26%)
Child increased risk of HD later	101 (54%)	28 (40%)
Most likely cause to HM in children is CHD	71 (38%)	16 (23%)

Abbreviations HM: heart murmur, HD: heart disease, CHD: congenital heart disease
